# Cardiac lipoma in a patient with a history of malignant tumours: a case report

**DOI:** 10.1186/1757-1626-1-41

**Published:** 2008-07-17

**Authors:** Bjoern Kitzing

**Affiliations:** 1 Westmead Hospital, Cnr Hawkesbury and Darcy Roads, Sydney, New South Wales, Australia

## Abstract

Cardiac tumours are relatively rare. Most of them do not cause clinical symptoms so that prior to the introduction of modern methods of investigation they were predominately incidental findings during autopsies or open heart surgery.

We present the case of a 63-year-old German man with a past history of malignant melanoma and renal cell carcinoma who presented with a right atrial lipoma and a suspicious lesion in the right lower lobe of the lung. Surgical excision of the cardiac lipoma was performed and a biopsy of the lung mass was done which diagnosed a moderately differentiated adenocarcinoma.

## Introduction

This is a rare case of a patient with a right atrial lipoma on a background of multiple malignant tumours who was admitted to hospital for excision of same. The diagnosis had been made incidentally following magnetic resonance imaging of the chest.

## Case presentation

A 63-year-old German man with a long past history of tumours was admitted to hospital. 20 years ago a malignant melanoma had been diagnosed on his right loin and radical excision performed. Due to lymph node metastasis in the left groin the patient had chemotherapy. After some time the patient developed haematuria whereupon renal cell carcinoma was diagnosed in the right kidney and a metastasis of the melanoma was found in the left kidney. Partial nephrectomy on the right and complete nephrectomy on the left were performed. The patient had since been free of tumour recurrence until presentation.

**Figure 1:**
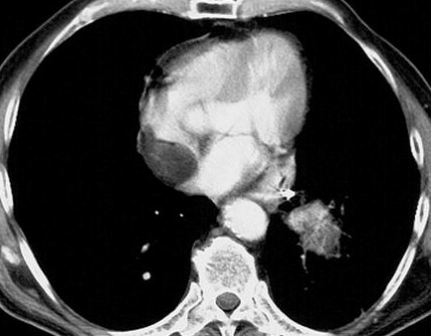
Select axial image of computed tomography showing a right atrial lipoma.

A recent chest X-ray  showed a suspicious shadow in the right lower lobe of the lung . Two computer tomography examinations of the chest (fig 1) were performed but could not conclusively rule out the possibility of metastasis. It was diagnosed initially as an inflammatory process of the lung. When the shadow failed to change with time further investigations were ordered. This was when a tumour in the right atrium was discovered which was neither haemodynamically nor clinically relevant. Magnetic resonance imaging of the chest showed a spherical tumour (28 × 27 × 20 mm) attached to the wall of the right atrium. The T1 weighted image revealed homogenous signal intensity as well as a loss of signal due to fat tissue. The tumour didn't show any signs of contrast medium uptake so that the diagnosis of a benign lipoma was made preoperatively. Transthoracic echocardiography excluded heart valve dysfunction and confirmed the presence of a mass in the right atrium.

On account of the above-mentioned criteria and of the absence of metastasis of the melanoma excision of the lipoma as well as a biopsy of the mass in the right lower lobe was planned. The patient was clinically stable. Heart rate, blood pressure, ECG and lab results were normal with the exception of urea & electrolytes. After careful preparations the lipoma excision was carried out through incision of the right atrium while the patient was supported by cardiopulmonary bypass. The tumour was walnut-sized, yellow, had a smooth surface and was attached to the lateral wall of the right atrium. The excision was performed at the base of the lipoma in the atrial wall. The atrium was then sutured. Afterwards, with the patient now being supported by partial cardiopulmonary bypass, the right lung was inspected. A hard resistance could be palpated in the right lower lobe. Considering the uncertainty of its nature and the use of extracorporeal circulation lobectomy was not performed. A biopsy was performed instead. The pleura was closed and the operation successfully completed.

The patient recovered well on the ward and was discharged 9 days after the operation with an appointment for a partial lobectomy at a different hospital. Macroscopical and histological examination of the cardiac tumour showed a tumour measuring 40 × 25 × 20 mm, grey-yellowish coloured on cross section with adequate excisional margin. It was attached to the atrial myocardium which showed signs of interstitial fibrosis. The mesenchymal tumour was capsulated and found to be of variable width and made up of unilocular uniform adipocytes with small nuclei and focal regressive changes as well as focal giant cell granulomas. Thus, the diagnosis lipoma was correct and a good prognosis can be expected. As for the lung tumour a moderately differentiated adenocarcinoma was diagnosed.

## Conclusion

Cardiac tumours are relatively rare. Most of them do not cause clinical symptoms so that prior to the introduction of modern methods of investigation they were predominately incidental findings during autopsies or open heart surgery. There are two different types of cardiac tumours: primary and secondary. Primary heart tumours can either be benign or malignant and make up about 0.0017 – 10% of all cardiac neoplasms [1,2]. Secondary tumours are 20 times more common. They represent metastases of end-stage malignancies and hence have less clinical significance. Of the primary heart tumours 75% are benign, only 25% malignant. The malignant tumours are characterized by aggressive and infiltrative growth into surrounding structures and are therefore difficult to treat surgically. The most common malignant cardiac neoplasms are sarcomas from a range of different tissues. The most common type of benign tumour in children is rhabdomyoma (ca. 20%) and in adults myxoma (ca. 50%). Cardiac lipomas are 50 times less common than myxomas and usually present in combination with lipomas of other organs.

As a benign tumour the lipoma has similar growth and spreading characteristics to the myxoma. However, it grows significantly slower. Macroscopically, they are often round and capsulated. They usually originate from epicardial fat tissue and grow into the pericardial sac. The most common intracardial localisation is in the right atrium with a wide peduncle originating either from the septal wall or atrial roof. They have also been described in the pericardium, left atrium, on the heart valves, in the pulmonary veins, in the right coronary artery as well as in the right and left ventricles. What all lipomas have in common is their proliferation pattern. Following a long asymptomatic period of about 30 – 40 years the lipomas begin to proliferate within their capsules and can cause a variety of different clinical symptoms. The patients concerned are often obese. The most common symptoms are dyspnoea, embolism, atrial and ventricular arrhythmias. Other symptoms that have been described are palpitations, ECG changes, angina pectoris, heart valve dysfunction, flow obstruction in the inferior or superior caval vein, cardiac failure as well as phrenic nerve lesions.

Lipomatous hypertrophy of the atrial septal wall is a condition not related to tumours but rather due to non-capsulated fat cells that infiltrate the interatrial wall which primarily effects obese patients over 50 years of age. These patients usually present with arrhythmias and treatment should consist of a change of diet.

If one suspects a patient has a cardiac tumour it can be diagnosed non-invasively using echocardiography. The information that echocardiography can provide includes tumour size, surface texture, localisation and mobility. Performing thoracoscopy for diagnostic purposes or biopsy collection has not proven beneficial. Angiography is an option if blood vessel supply is of interest or to exclude other cardiac diseases. If pericardial effusion is present pericardiocentesis and cytological examination of the pericardial fluids should be performed. Computer tomography or magnetic resonance imaging is also helpful as it yields not only morphological information but also precise information about the density of the tumour. In the case where the tumour's density is equivalent to that of subcutaneous tissue the preoperative diagnosis of a cardiac lipoma can be made.

Genetic studies of cardiac lipomas have also been carried out as it has often been assumed that these tumours have similar chromosomal variations as common subcutaneous lipomas. However, a relevant connection could not be shown.

Histologically, cardiac lipomas have the same structure as other lipomas but are always capsulated. Microscopically, it is made up of mature fat cells and streaks of fibrous tissue with central necrosis [2].

Long-term follow-up studies of patients with benign tumours show an extremely good outcome in patients who have radical surgery with no tumour recurrence whereas malignant tumours have a bad prognosis on account of their invasive growth and early metastasizing.

The operation of choice depends on the localisation and size of the lipoma. Current literature recommends that even asymptomatic patients should be operated on soon after the diagnosis has been made irrespective of size and speed of growth [3,4]. Symptomatic patients should have urgent surgical treatment.

To conclude, this is a rare case of a patient with a history of malignant melanoma and renal cell carcinoma presenting for elective surgery of a cardiac lipoma who was subsequently diagnosed to have an adenocarcinoma of the lung. Considering the fact that there was nothing in the history to account for the unusual occurrence of these tumours one might suspect an underlying genetic disposition.

## List of abbreviations

CT: Computed tomography; ECG: Electrocardiogram; MRI: Magnetic resonance imaging.

## Consent

Written informed consent was obtained from the patient for publication of this case report. A copy of the written consent is available for review by the Editor-in-Chief of this journal.

## Competing interests

The author declares that they have no competing interests.

## Authors' contributions

BK made substantial contributions to conception and design, drafted the manuscript, revised it critically for important intellectual content and gave final approval of the version to be published.
